# CsMLO8/11 are required for full susceptibility of cucumber stem to powdery mildew and interact with CsCRK2 and CsRbohD

**DOI:** 10.1093/hr/uhad295

**Published:** 2023-12-29

**Authors:** Shaoyun Dong, Xin Liu, Jianan Han, Han Miao, Diane M Beckles, Yuling Bai, Xiaoping Liu, Jiantao Guan, Ruizhen Yang, Xingfang Gu, Jiaqiang Sun, Xueyong Yang, Shengping Zhang

**Affiliations:** State Key Laboratory of Vegetable Biobreeding, Institute of Vegetables and Flowers, Chinese Academy of Agricultural Sciences, 100081, Beijing China; State Key Laboratory of Vegetable Biobreeding, Institute of Vegetables and Flowers, Chinese Academy of Agricultural Sciences, 100081, Beijing China; State Key Laboratory of Vegetable Biobreeding, Institute of Vegetables and Flowers, Chinese Academy of Agricultural Sciences, 100081, Beijing China; State Key Laboratory of Vegetable Biobreeding, Institute of Vegetables and Flowers, Chinese Academy of Agricultural Sciences, 100081, Beijing China; Department of Plant Sciences, University of California Davis, One Shield Avenue, Davis, CA 95616, USA; Plant Breeding, Wageningen University & Research, Droevendaalsesteeg 1, 6708 PB Wageningen, The Netherlands; State Key Laboratory of Vegetable Biobreeding, Institute of Vegetables and Flowers, Chinese Academy of Agricultural Sciences, 100081, Beijing China; State Key Laboratory of Vegetable Biobreeding, Institute of Vegetables and Flowers, Chinese Academy of Agricultural Sciences, 100081, Beijing China; Institute of Crop Sciences, Chinese Academy of Agricultural Sciences, 100081 Beijing, China; State Key Laboratory of Vegetable Biobreeding, Institute of Vegetables and Flowers, Chinese Academy of Agricultural Sciences, 100081, Beijing China; Institute of Crop Sciences, Chinese Academy of Agricultural Sciences, 100081 Beijing, China; State Key Laboratory of Vegetable Biobreeding, Institute of Vegetables and Flowers, Chinese Academy of Agricultural Sciences, 100081, Beijing China; State Key Laboratory of Vegetable Biobreeding, Institute of Vegetables and Flowers, Chinese Academy of Agricultural Sciences, 100081, Beijing China

## Abstract

Powdery mildew (PM) is one of the most destructive diseases that threaten cucumber production globally. Efficient breeding of novel PM-resistant cultivars will require a robust understanding of the molecular mechanisms of cucumber resistance against PM. Using a genome-wide association study, we detected a locus significantly correlated with PM resistance in cucumber stem, *pm-s5.1*. A 1449-bp insertion in the *CsMLO8* coding region at the *pm-s5.1* locus resulted in enhanced stem PM resistance. Knockout mutants of *CsMLO8* and *CsMLO11* generated by CRISPR/Cas9 both showed improved PM resistance in the stem, hypocotyl, and leaves, and the double mutant *mlo8mlo11* displayed even stronger resistance. We found that reactive oxygen species (ROS) accumulation was higher in the stem of these mutants. Protein interaction assays suggested that CsMLO8 and CsMLO11 could physically interact with CsRbohD and CsCRK2, respectively. Further, we showed that CsMLO8 and CsCRK2 competitively interact with the C-terminus of CsRbohD to affect CsCRK2-CsRbohD module-mediated ROS production during PM defense. These findings provide new insights into the understanding of CsMLO proteins during PM defense responses.

## Introduction

Powdery mildew (PM) is one of the most common and widespread diseases in cucumber (*Cucumis sativus* L.) [1]. PM is caused by the fungus *Podosphaera fusca,* and the symptoms are characterized by small, white, powdery fungal growth on stems and leaves, leading to leaf shrinkage, premature senescence, and death [2]. At present, the application of fungicides is the primary way to control PM in commercial agricultural production. However, PM pathogens often acquire resistance to fungicides, rendering them ineffective, while these chemicals remain harmful to human health and to the environment [3]. Breeding PM-resistant cucumber cultivars is the most efficient and environmentally friendly method to control PM.

PM-resistant cucumber lines have been reported since the 1940s, mostly from Asian germplasm. They include ‘Puerto Rico 37’ from Chinese germplasm [4], PI 197087 from India [5], ‘Yomaki’ (PI288238) and ‘Natsufushinari’ (PI 279465) from Japan [6], and ‘Bangalore’ and ‘Burma’ lines (PI 200815 and PI 200818) from India [4]. These germplasms and their derived lines have been used for subsequent cucumber PM resistance breeding. Resistance in these accessions was primarily due to the presence of multiple recessive genes.

Quantitative trait loci (QTLs) for PM resistance in cucumber leaves have been detected on all seven cucumber chromosomes. Sakata *et al*. [7] first reported QTLs for PM resistance using 95 cucumber accessions; six QTLs underlying PM resistance in the Indian accession PI197088-1 were detected, including one major QTL conferring resistance. Liu *et al*. [8] identified five QTLs responsible for PM resistance originating from a European greenhouse cucumber line, ‘S06’. Zhang *et al*. [9] detected four QTLs (*pm5.1*, *pm5.2*, *pm5.3*, and *pm6.1*) controlling PM resistance in North China cucumber line ‘K8’ and identified a major QTL on chromosome 5. He *et al*. [10] detected four QTLs (*pm-tl1.1*, *pm-tl1.2*, *pm-tl5.1*, and *pm-tl5.2*) for PM resistance on the true leaf of ‘WI 2757’. Nie *et al*. [11] detected a major QTL, *pm5.1*, in an East Asian line, ‘S1003’. Moreover, Zhang *et al*. [12] detected two major QTLs on chromosomes 1 and 6 using ‘BK2’ and ‘H136’. *Wang et al*. [13] identified four QTLs (*pm1.1*, *pm2.1*, *pm5.1*, *pm 6.1*) in PI197088. *Liu et al*. [14] performed a genome-wide association study (GWAS) on leaf PM resistance and detected 13 PM loci distributed across almost all chromosomes.

While many QTLs for leaf PM resistance have been identified, only a few candidate genes underlying these QTLs have been predicted. Xu *et al*. [15] identified *Csa1M064780* and *Csa1M064790*, encoding cysteine-rich receptor-like kinases, as candidate genes. Zhang *et al*. [16] suggested that *Csa5M622830*, which encodes a GATA transcription factor, was the most likely candidate gene. Recently, Zhang *et al*. [17] suggested that *CsGy5G015660*, which encodes a putative leucine-rich repeat receptor-like serine/threonine-protein kinase, was the causal gene for PM resistance.

To date, very few studies have focused on PM resistance on cucumber stems or hypocotyls. He *et al*. [10] detected three QTLs (*pm-hy3.1*, *pm-hy4.1*, and *pm-hy5.1*) for hypocotyl resistance in ‘WI 2757’, with *pm-hy5.1* playing a key role. Liu *et al*. [18] found a recessive nuclear gene (*pm-s*) controlling stem PM resistance in ‘NCG-122’, and predicted that the Mildew Resistance Locus O (MLO)-related protein-encoding gene *Csa5G623470* as the most possible candidate gene. However, the function of these genes has not yet been verified via genetic complementation studies, and therefore the genetic and molecular mechanisms underlying PM resistance remain unclear.

S-genes have been the focus of many plant–pathogen interaction studies because they encode proteins that facilitate host recognition and defense suppression, and enable pathogen penetration of plant tissue [19]. Loss of S-gene function confers broad-spectrum resistance against a variety of diseases in plants [20]. Over 180 S-genes have been identified in various plant species [19]. Among them, *Mildew Resistance Locus O *(*MLO*), which encodes a plasma membrane-anchored protein, is the most widely known S-gene responsible for PM susceptibility in plants [21]. Resistance mediated by *mlo* was first discovered in barley [22], and since then, *mlo*-mediated resistance to PM has been reported in other plant species, e.g. *AtMLO2*, *AtMLO6*, and *AtMLO12* in *Arabidopsis* [23], *SlMLO1* in tomato [24], *TaMLO_A1* and *TaMLO_B1* in wheat [25], *CaMLO2* in pepper [26], *VvMLO3*, *VvMLO6*, and *VvMLO7* in grapevine [27–29], *FaMLO10* and *FaMLO20* in strawberry [30], and *PsMLO1* in pea [31, 32]. In cucumber, 16 *CsMLO* genes have been identified, and three genes that belong to clade V, i.e. *CsMLO1*, *CsMLO8*, and *CsMLO11*, were associated with PM susceptibility [33]. *CsMLO8* was co-located with a major-effect recessive QTL on chromosome 5 for leaf and hypocotyl PM resistance in several studies [10, 11, 18, 34], while *CsMLO1* and *CsMLO11* were co-localized with QTLs *pm1.1* and *pm6.1*, respectively [35]. While some studies showed that *mlo*-mediated resistance by plants depends on enhanced callose deposition [36] and increased defense responses such as hydrogen peroxide bursts [37], the molecular mechanism underlying this defense response is still ambiguous, especially in cucumber.

In this study we performed a GWAS using 95 diverse cucumber accessions in two seasons, and detected a major locus significantly associated with stem PM resistance. The candidate gene *CsMLO8* within the *pm-s5.1* locus, and its homolog *CsMLO11*, were functionally characterized. Mutants of *mlo8*, *mlo11*, and *mlo8mlo11* showed improved PM resistance. In addition, the accumulation of reactive oxygen species (ROS) increased in the stems of these mutants. We further demonstrated that CsMLO8 and CsMLO11 could physically interact with respiratory burst oxidase homolog D (CsRbohD) and cysteine-rich receptor-like kinase 2 (CsCRK2), respectively. Further, CsMLO8 and CsCRK2 competitively interact with the C-terminus of CsRbohD to regulate ROS production during PM defense. These findings provide new insights into the function of the *CsMLO* genes for PM defense responses.

## Results

### Genetic diversity of powdery mildew resistance among accessions of cucumber core germplasms

**Figure 1:**
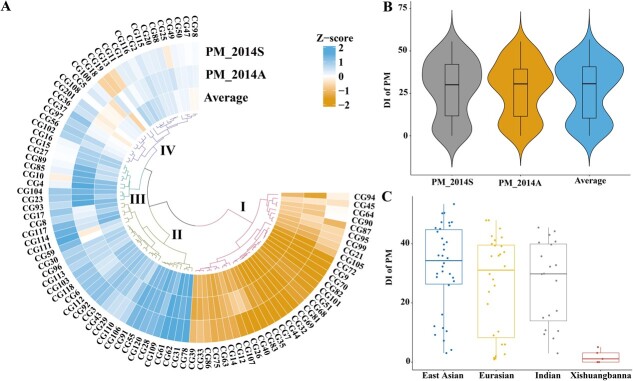
Genetic diversity of stem PM resistance among accessions of cucumber core germplasms. **a** Heat map indicating the DI of stem PM resistance in spring 2014 (PM_2014S) and fall 2014 (PM_2014A). The four clusters of the CG accessions are numbered I–IV. Yellow means resistant and blue susceptible. **b** Violin plots depicting the distribution of DI in two seasons. **c** Box plots of DI of PM among different ecotypes.

A collection of 95 accessions from cucumber core germplasm (CG), acquired from different regions of the world (Supplementary Data Table S1), was characterized for PM resistance in stems in spring 2014 and fall 2014. The average disease index (DI) of each accession was calculated in each experiment (Fig. 1a, Supplementary Data Table S1). PM resistance showed diverse phenotypic variations, ranging from 0 to 55.56 in both the spring and fall trials (Fig. 1b; Supplementary Data Table S2). The cucumber accessions were classified into four clusters based on the average DI: I, highly tolerant; II, tolerant; III, sensitive; and IV, highly sensitive (Fig. 1a; Supplementary Data Table S3). A total of 33 highly tolerant germplasms with DIs ranging from 0.00 to 20.99 and seven highly sensitive germplasms with DIs ranging from 46.28 to 53.09 were identified. The 95 accessions consisted of four ecotypes, including East Asian (*n* = 35 lines), Eurasian (*n* = 29), Indian (*n* = 21), and Xishuangbanna (*n* = 10) types (Fig. 1c; Supplementary Data Table S1). The mean DI of PM in the Xishuangbanna ecotype was lower than all others, suggesting that the Xishuangbanna ecotype is more resistant to PM infection (Fig. 1c).

### Genome-wide association studies reveal *CsMLO8* as a candidate gene for stem powdery mildew resistance

**Figure 2:**
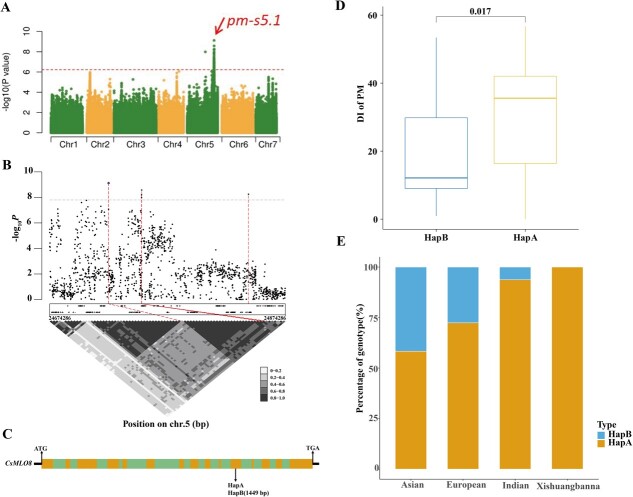
GWASs reveal that natural variation of *CsMLO8* confers PM resistance in cucumber stem**. a** GWAS Manhattan plot for stem PM resistance in spring of 2014. The genome-wide significance threshold was *P* = 5.9 × 10^−7^. The red arrow indicates the position of a strong peak (*pm-s5.1*). **b** The LD block surrounding the *pm-s5.1* peak. The red dotted lines indicate the candidate region. **c** Gene structure of *Csa5G623470* in the interval. Filled yellow box, filled green box, and black line represent exons, introns, and the UTR respectively. **d** Box plots of DI, based on the haplotypes of *Csa5G623470*: HapA (WT) and HapB (with a 1449-bp insertion). **e** Proportion of HapA and HapB alleles within each ecotype.

To identify the key genes for PM resistance on cucumber stem, the DI of each experiment was used for a GWAS using a mixed linear model. A stable association signal in a 200-kb region on chromosome 5 was detected across the two seasons and was named *pm-s5.1* (Fig. 2a; Supplementary Data Fig. S1; Supplementary Data Table S4). To identify potential candidate genes within this locus, SNPs of chromosome 5 between nucleotides 24 674 286 and 24 874 286 were analyzed by pairwise linkage disequilibrium (LD) correlations (Fig. 2b). We then focused on the interval from 24 724 286 to 24 842 958 nucleotides (*r*^2^ ≥ 0.6). There are 19 annotated ‘protein-coding’ genes in this region, using the cucumber ‘9930’ reference genome (Supplementary Data Table S5). Among these genes, *Csa5G623470* was of interest; it encoded a seven-transmembrane-domain protein and there was a 1449-bp insertion (HapB) in the 11th exon (Fig. 2c). The HapB accessions all had significantly (*P* = 0.017) lower DIs than those without the insertion (HapA types) (Fig. 2d), indicating that *Csa5G623470* is a candidate gene for PM resistance in cucumber stem. Interestingly, the HapB allele existed in the Eurasian, East Asian, and Indian types, but was absent in the Xishuangbanna-type cucumbers (Fig. 2e), suggesting that the strong resistance seen on the stems of the 10 Xishuangbanna-type accessions is not caused by the loss of *CsMLO8*.

### Mutations in *CsMLO8* and *CsMLO11* confer resistance of the relevant mutants to powdery mildew

**Figure 3:**
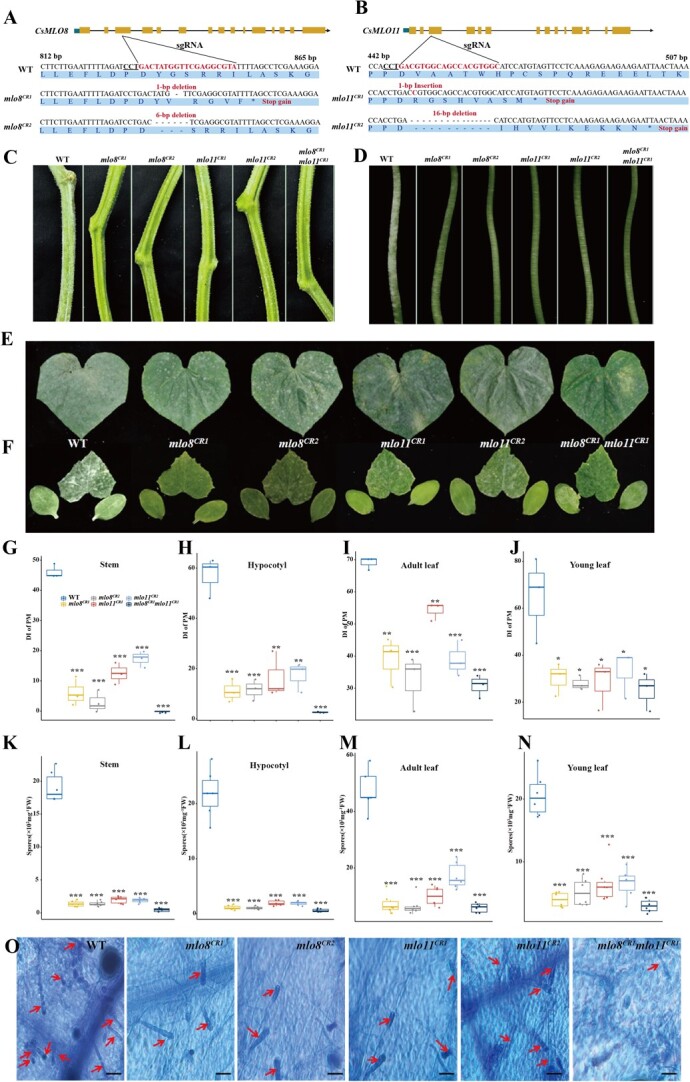
PM resistance in CRISPR/Cas9 mutants of *CsMLO8/11****.* a**, **b** Schematic illustrating the gene structure of (**a**) *CsMLO8* and (**b**) *CsMLO11*; filled blue, yellow boxes, and black lines represent the UTR, exons, and introns respectively. *mlo8^CR1^* and *mlo8^CR2^* are homozygous mutants for *CsMLO8. mlo11^CR1^* and *mlo11^CR2^* are homozygous mutants for *CsMLO11*. **c**–**f** PM infection phenotype of mutants (*mlo8^CR1^*, *mlo8^CR2^*, *mlo11^CR1^*, *mlo11^CR2^*, *mlo8^CR1^mlo11^CR1^*) and WT plants in (**c**) stem, (**d**) hypocotyl, (**e**) adult leaf, and (**f**) young leaf. Photographs were taken at 62 dps for the stem, 8 dpi for the hypocotyl, 55 dps for the adult leaves, and 6 dpi for the young leaves. **g*–*j** Statistical analyses of DI for (**g**) stem, (**h**) hypocotyl, (**i**) adult leaf, and (**j**) young leaf. ^***^P < 0.001; ^**^P < 0.01; ^*^P < 0.05, Student’s *t*-test, *n* = 3. **k**–**n** Number of spores in (**k**) stem, (**l**) hypocotyl, (**m**) adult leaf, and (**n**) young leaf. ***P < 0.001, Student’s *t*-test, *n* = 6. **o** Trypan blue staining of young leaves of the WT and *mlo* mutants at 6 dpi. Red arrows indicate spore hyphae. Scale bars, 50 μm.

To validate the function of *CsMLO8* in PM resistance, we knocked out *CsMLO8* in ‘CU2’, a variety harboring the HapA allele, which shows PM susceptibility on the stem, using the CRISPR/Cas9 system. Two independent homozygous knockout lines, named *mlo8^CR1^* and *mlo8^CR2^*, respectively, were obtained. *mlo8^CR1^* had a 1-bp deletion which led to an early stop codon, while *mlo8^CR2^* had a 6-bp deletion on the third exon. These deletions are predicted to produce polypeptides with a 446- and 2-amino acid deletion in *mlo8^CR1^* and *mlo8^CR2^*, respectively (Fig. 3a). Since *CsMLO11* has been previously reported as a PM-susceptible gene, we also knocked out *CsMLO11* in ‘CU2’. Two independent homozygous knockout lines, named *mlo11^CR1^* and *mlo11^CR2^*, respectively, were obtained; *mlo11^CR1^* had a 1-bp insertion while *mlo11^CR2^* had a 16-bp deletion in the third exon, and both polymorphisms led to the creation of an early stop codon (Fig. 3b). A double mutant of *CsMLO8* and *CsMLO11* (*mlo8^CR1^mlo11^CR1^*) was also generated by crossing the single mutants.

These mutants (*mlo8^CR1^*, *mlo8^CR2^*, *mlo11^CR1^*, *mlo11^CR2^*, and *mlo8^CR1^mlo11^CR1^*) together with the wild-type control (WT) were evaluated for PM resistance at the seedling and adult stages. For adult stem, at 62 days after sowing (dps), the WT showed severe PM infection with an average DI of 46.2. The *mlo8^CR1^* and *mlo8^CR2^* genotypes both showed significantly increased stem PM resistance, with a DI of 6.0 and 3.0, respectively. The *mlo11^CR1^* and *mlo11^CR2^* genotypes also showed significantly reduced stem PM infection, with a DI of 12.6 and 17.4, respectively. For the double mutant *mlo8^CR1^mlo11^CR1^*, no PM infection was detected on the stem (Fig. 3c and g). For hypocotyls, the WT showed severe PM infection at 8 days post-inoculation (dpi), with an average DI of 57.1. However, *mlo8^CR1^* and *mlo8^CR2^* both showed significantly increased PM resistance, with a DI of 11.0 and 11.7, respectively. The *mlo11^CR1^* and *mlo11^CR2^* genotypes also showed significantly reduced PM infection, with a DI of 16.50 and 17.3, respectively. The *mlo8^CR1^mlo11^CR1^* double mutant exhibited the strongest resistance, with a DI of 2.7 (Fig. 3d and h). We also evaluated PM resistance in young and adult leaves. At 55 dps, the adult leaf of the WT showed severe PM symptoms, with an average DI of 69, while the *mlo* mutants were less affected, with DIs of 32.5–39 in *mlo8*, 39–54.2 in *mlo11*, and 30.9 in *mlo8^CR1^mlo11^CR1^* (Fig. 3e and i). At 6 dpi, the first true leaf of the WT showed severe disease symptoms, while symptoms were mild in the mutants. The average DI of the WT was 65, and was significantly higher than those of *mlo8* (30.2, 28), *mlo11* (28.5, 33.2), and *mlo8^CR1^mlo11^CR1^* (25.1) mutants (Fig. 3f and j). Consistent with this observation, compared with the WT control, *mlo8*, *mlo11*, and *mlo8^CR1^mlo11^CR1^* had 13-, 9-, and 42-fold fewer spores on the infected stems at 62 dps (Fig. 3k), and 20-, 10-, and 35-fold fewer spores on hypocotyls at 8 dpi, respectively (Fig. 3l). In addition, *mlo8, mlo11*, and *mlo8^CR1^mlo11^CR1^* had 6-, 4-, and 7-fold fewer spores on infected adult leaves at 55 dps (Fig. 3m), and 4-, 2-, and 6-fold fewer spores on young leaves at 6 dpi, respectively (Fig. 3n). Moreover, Trypan blue staining revealed that there were fewer fungal hyphae and conidia in the mutants compared with the WT at 6 dpi (Fig. 3o). These results indicate that disruption of *CsMLO8* and/or *CsMLO11* results in enhanced resistance to PM, and the resistance is more obvious in the stem and hypocotyl.

### Multiple metabolism- and defense-related genes were upregulated in the *CsMLO8/11* mutants

To further investigate the molecular mechanism by which *CsMLO8* and *CsMLO11* regulate PM resistance in cucumber, we compared the transcriptomic profiles of mutant (*mlo8^CR1^*, *mlo11^CR1^*, *mlo8^CR1^mlo11^CR1^*) and WT hypocotyls before inoculation (0 hpi) and 24 h post-inoculation (24 hpi).

**Figure 4:**
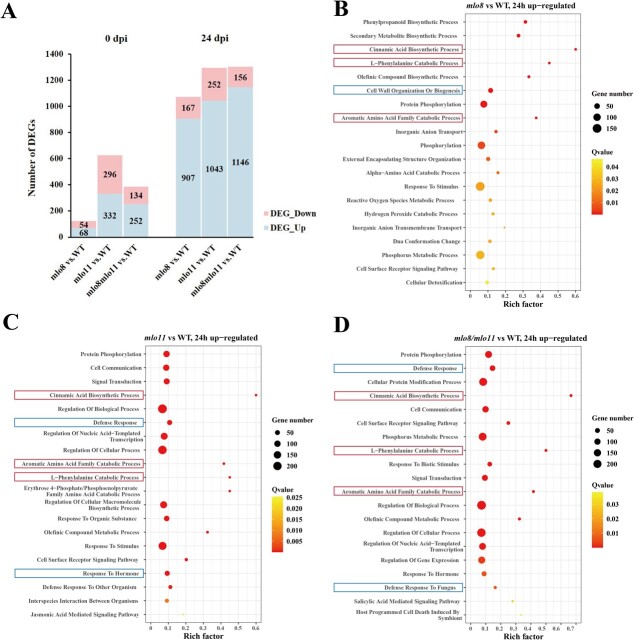
DEGs between *mlo8^CR1^* and *mlo11^CR1^* CRISPR-Cas9 mutant lines and controls. **a** Up- and downregulated DEGs in *mlo8^CR1^*, *mlo11^CR1^*, and *mlo8^CR1^mlo11^CR1^* relative to WT control at 0 and 24 hpi. GO pathways enriched among upregulated genes of (**b**) *mlo8^CR1^* vs WT, (**c**) *mlo11^CR1^* vs WT, and (**d**) *mlo8^CR1^mlo11^CR1^* vs WT. The red boxes indicate the common pathways detected in the three tested mutants, while the blue boxes represent the unique pathways found in the different mutants.

Before inoculation, a total of 122 differentially expressed genes (DEGs) were identified between *mlo8^CR1^* and the WT, and 628 and 386 DEGs between the WT and *mlo11^CR1^* and *mlo8^CR1^mlo11^CR1^*, respectively (Fig. 4a). Interestingly, at 24 hpi the number of DEGs was significantly increased compared with 0 hpi: 1074 in *mlo8^CR1^*, 1295 in *mlo11^CR1^*, and 1302 in *mlo8^CR1^mlo11^CR1^* compared with the WT. Among them, 84.5, 80.5, and 88.0% of the DEGs were upregulated in *mlo8^CR1^*, *mlo11^CR1^*, and *mlo8^CR1^mlo11^CR1^*, respectively (Fig. 4a).

To better understand the DEGs in the aforementioned mutants at 24 hpi, Gene Ontology (GO) enrichment was performed. Several significantly upregulated pathways (*q* value <0.05) related to plant disease resistance were enriched. Primary and secondary metabolites, including l-phenylalanine, aromatic amino acid, and cinnamic acid, were also upregulated in *mlo8^CR1^*, *mlo11^CR1^*, and *mlo8^CR1^mlo11^CR1^* (Fig. 4b–d; Supplementary Data Table S6)*.* Interestingly, pathways related to cell wall organization or biogenesis were uniquely enhanced in *mlo8^CR1^* (Fig. 4b), while pathways related to defense response were significantly upregulated in *mlo11^CR1^* and *mlo8^CR1^mlo11^CR1^* (Fig. 4c and d). These results indicated that *CsMLO8* and *CsMLO11* may function in overlapping cellular signaling pathways.

### More reactive oxygen species accumulation in *mlo8/11* stem after powdery mildew inoculation

**Figure 5:**
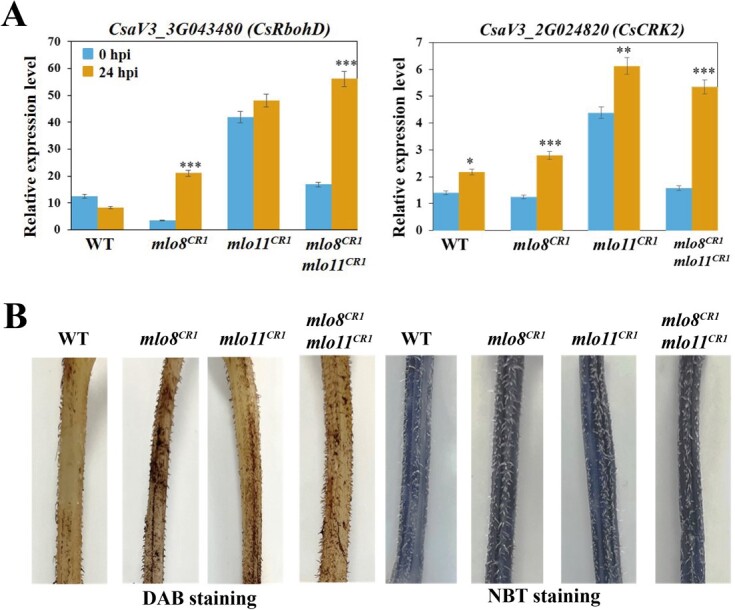
Changes in ROS accumulation in stems of *CsMLO* mutants and WT after PM infection**. a** Relative expression levels of *CsRbohD* and *CsCRK2* at 0 and 24 hpi ^*^P < 0.05, ^**^P < 0.01, ^***^P < 0.001, ANOVA, Student’s *t*-test. **b** Stems harvested at 6 dpi were stained. Blue and brown precipitate indicates O_2_^−^ and H_2_O_2_ location and content.

ROS production is important in plant pathogen-defense responses. NADPH oxidase/respiratory burst oxidase homolog (Rboh) proteins function in localized ROS ‘bursts’ [38]. RbohD is required for ROS production during the immune response in *Arabidopsis* [39]. CYSTEINE-RICH RLK2 (CRK2) kinase could interact with RbohD and is required for the full elicitor-induced ROS burst [40]. Interestingly, we found that *CsaV3_3G043480* (*CsRbohD*) was significantly upregulated by PM inoculation in *mlo8^CR1^*, and *mlo8^CR1^mlo11^CR1^*, but not in the WT controls. Moreover, the expression level of *CsaV3_2G024820* (*CsCRK2*) increased in WT, *mlo8^CR1^*, *mlo11^CR1^*, and *mlo8^CR1^mlo11^CR1^* after PM inoculation, and the upregulation was more significant in *mlo8^CR1^* and *mlo8^CR1^mlo11^CR1^* (Fig. 5a; Supplementary Data Table S7). Moreover, from the RNA-seq data, we found that the transcripts of genes encoding key antioxidant enzymes involved in ROS scavenging, e.g. superoxide dismutase (SOD), glutathione peroxidase (GPX), ascorbate peroxidase (APX), and glutathione *S*-transferase (GST), were decreased in WT, *mlo8^CR1^*, *mlo11^CR1^*, and *mlo8^CR1^mlo11^CR1^* at 24 hpi compared with 0 hpi (Supplementary Data Table S7).

To investigate whether the enhanced stem PM resistance of *CsMLO* mutants was related to ROS accumulation, we examined hydrogen peroxide (H_2_O_2_) and superoxide anions (O_2_^−^) in PM-inoculated stems. Diaminobenzidine (DAB) and nitroblue tetrazolium (NBT) staining revealed a higher increase in H_2_O_2_ and O_2_^−^ accumulation, respectively, in the stem of the three mutants compared with control plants, suggesting that *mlo8^CR1^*, *mlo11^CR1^*, and *mlo8^CR1^mlo11^CR1^* conferred increased ROS production (Fig. 5b). These results suggested that dysfunction of the *CsMLO8* and *CsMLO11* genes facilitates a burst of ROS production which contributes to the PM immune response.

### CsMLO8 and CsMLO11 could physically interact with CsRbohD and CsCRK2

**Figure 6:**
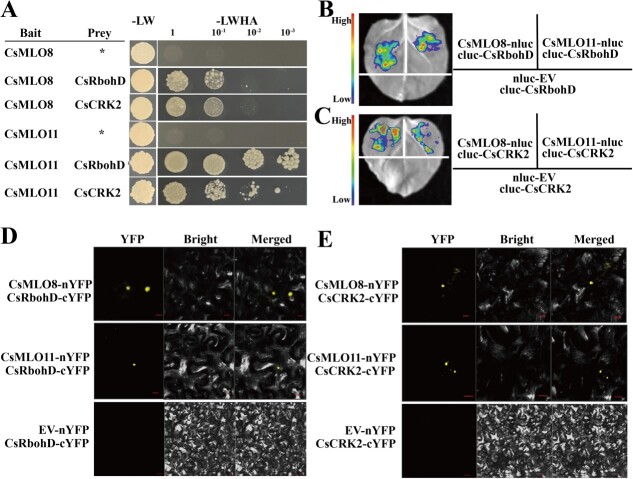
Physical interaction assays for CsMLO8/11, CsRbohD, and CsCRK2. **a** Y2H analyses showing the interaction between CsMLO8/11 and CsRbohD and between CsMLO8/11 and CsCRK2. Asterisks indicate empty vectors. Positive interactions were tested using yeast cells grown on synthetic defined medium lacking Leu, Trp, His, and adenine (−LWHA). **b**, **c** LCI assays showing the interaction between (**b**) CsMLO8/11 and CsRbohD, and (**c**) CsMLO8/11 and CsCRK2. **d**, **e** BiFC assays showing the interaction between (**d**) CsMLO8/11 and CsRbohD, and (**e**) CsMLO8/11 and CsCRK2. Scale bars, 10 μm.

To further investigate whether CsMLO8 or CsMLO11 could physically interact with CsRbohD and CsCRK2, we carried out diverse biochemical assays. First, yeast two-hybrid (Y2H) assays demonstrated that CsMLO8 or CsMLO11 indeed interacts with CsRbohD and CsCRK2 (Fig. 6a). To further confirm the protein interactions in plant cells, we performed luciferase complementation imaging assays (LCIs) in *Nicotiana benthamiana* leaves. CsMLO8 and CsMLO11 were fused to the N-terminus of LUC (nLUC) to generate CsMLO8-nLUC and CsMLO11-nLUC, respectively; meanwhile, CsRbohD and CsCRK2 were fused to the C-terminus of LUC (cLUC) to generate cLUC-CsRbohD and cLUC-CsCRK2. As revealed in Fig. 6b, strong LUC activities were detected in the samples co-expressing CsMLO8-nLUC/cLUC-CsRbohD and CsMLO11-nLUC/cLUC-CsRbohD. Similarly in Fig. 6c, strong LUC activities were detected in the samples co-expressing CsMLO8-nLUC/cLUC-CsCRK2 and CsMLO11-nLUC/cLUC-CsCRK2. We then performed transient bimolecular fluorescence complementation (BiFC) assays in *N. benthamiana* leaves. CsRbohD and CsCRK2 were fused to the C-terminus of yellow fluorescent protein (cYFP). CsMLO8 and CsMLO11 were fused to the N-terminus of YFP (nYFP). Strong interaction signals were detected in the CsMLO8-nYFP/CsRbohD-cYFP, CsMLO11-nYFP/CsRbohD-cYFP, CsMLO8-nYFP/CsCRK2-cYFP, and CsMLO11-nYFP/CsCRK2-cYFP co-expressing samples (Fig. 6d and e). Taking these results together, we concluded that CsMLO8/11 interacts with CsRbohD and CsCRK2.

### CsMLO8 and CsCRK2 competitively interact with the C-terminus of CsRbohD

**Figure 7:**
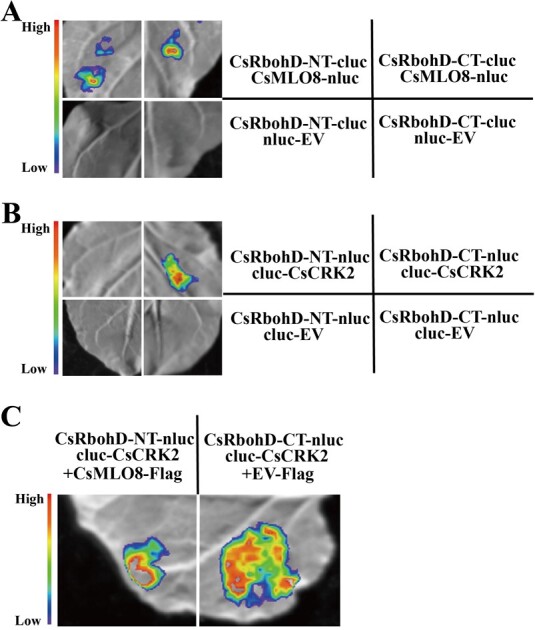
CsMLO8 interferes with the interaction of CsCRK2 and CsRbohD. **a**, **b** LCI assays showing the interaction between the truncated versions of CsRbohD and (**a**) CsMLO8 and (**b**) CsCRK2. **c** CsMLO8 inhibits the interaction of CsCRK2 and the C-terminus of CsRbohD. CsRbohD-NT, amino terminal domain of CsRbohD; CsRbohD-CT, carboxyl terminal domain of CsRbohD. *pCAMBIA1300*-flag was used as a control (EV-Flag) instead of CsMLO8-Flag. *Agrobacterium tumefaciens* strain GV3101 transformed with equal quantities of these constructs was injected into *N. benthamiana* leaves.

To determine which region of CsRbohD mediates the CsRbohD–CsMLO8 interaction, the N- and C-termini of CsRbohD were fused with cLUC, respectively, for LCI assays in *N. benthamiana* leaves. Obvious LUC activities were observed in both the CsMLO8-nLUC/cLUC-CsRbohD-CT and CsMLO8-nLUC/cLUC-CsRbohD-NT co-expression samples, but no LUC activity was observed in the control samples (Fig. 7a). These results suggest that CsMLO8 interacts with both the N- and C-termini of CsRbohD. Furthermore, it has been reported that CRK2 contributes to the activation of RbohD via phosphorylation of its C-terminus in *Arabidopsis* [40]. Our experiment (Fig. 7b) also showed LUC activities in the CsRbohD-CT-nLUC/cLUC-CsCRK2, but not in the CsRbohD-NT- nLUC/cLUC-CsCRK2 co-expression samples, demonstrating that CsCRK2 interacts with the C-terminus of CsRbohD. As both CsMLO8 and CsCRK2 interact with the C-terminus of CsRbohD, we wondered if CsMLO8 competitively interferes with the interaction between CsRbohD and CsCRK2. We therefore performed LCI assays in *N. benthamiana* leaves. As shown in Fig. 7c, the luminescence signals of the samples co-expressing CsRbohD-CT-nLUC/cLUC-CsCRK2 and CsMLO8-flag were significantly inhibited compared with those of samples co-expressing CsRbohD-CT-nLUC/cLUC-CsCRK2 and control vector. Taken together, these results indicate that CsMLO8 and CsCRK2 competitively interact with the C-terminus of CsRbohD.

## Discussion

PM is one of the most severe diseases that threaten cucumber production globally; breeding PM-resistant cultivars enables sustainable cucumber cultivation. However, the genes responsible for PM resistance and their underlying molecular mechanism in cucumber are relatively unknown. Here, we characterized PM resistance on the stems of 95 accessions of the core collection, which is a subset of the 3000 globally acquired cucumber accessions. Using 1 702 257 SNPs, we detected one locus, *pm-s5.1*, significantly associated with stem PM resistance on chromosome 5 in two seasons. The stem resistance conferred by *pm-s5.1* in this study is co-localized with a QTL, *pm-s*, for stem PM resistance we previously identified [18] and a QTL, *pm5.2*, for hypocotyl resistance detected by He *et al*. [10] . In addition, we had previously characterized PM resistance in the leaves of individuals from the core accessions [14], and identified 13 loci associated with leaf PM resistance. Among them, the *pmG5.4* locus co-localized with *pm-s5.1*, indicating that the major locus *pm-s5.1* confers both stem and leaf PM resistance. Compared with the loci for stem resistance, more QTLs for leaf resistance were detected across all seven chromosomes [7, 8, 10–14], suggesting that there are more genes involved in leaf PM resistance compared with stem PM resistance in cucumber.

Within the *pm-s5.1* locus, we found a 1449-bp insertion in the coding region of *CsMLO8* that resulted in significantly higher PM resistance on the stem. The result is consistent with the study by Berg *et al*. [34], who showed that a non-autonomous Class LTR retrotransposable element in *CsMLO8* was present frequently in cultivated cucumber germplasm, and caused hypocotyl PM resistance. Moreover, in our study, the Xishuangbanna ecotype generally had higher PM resistance in the stem compared with the other ecotypes. However, the geographical distribution of the HapA and HapB alleles, associated with susceptibility and resistance, respectively, showed that the HapB allele is absent among Xishuangbanna ecotypes, suggesting that it is not *CsMLO8* but another gene that is responsible for the stem PM resistance of Xishuangbanna-type cucumbers.

Among the 16 *CsMLO* genes in cucumber, *CsMLO1*, *CsMLO8*, and *CsMLO11* were associated with PM susceptibility [33]. The heterologous overexpression of *CsMLO8* in a tomato *mlo* mutant restored PM susceptibility [34]. Later studies showed that the overexpression of *CsMLO1* in the tomato *mlo* mutant completely restored PM susceptibility, while overexpression of *CsMLO11* only led to partial restoration [33]. *CsMLO1*, *CsMLO8*, and *CsMLO11* mutants were recently generated in cucumber plants [41]; the PM symptoms on *mlo8mlo11* and *mlo1mlo11* leaves were significantly decreased compared with *mlo1* plants, and no PM symptoms appeared on *mlo1mlo8mlo11* leaves [41]. However, PM resistance on stem or hypocotyl was not characterized in these studies. To verify the role of *CsMLO8* in stem resistance, we generated two independent *CsMLO8* knockout lines (named *mlo8^CR1^* and *mlo8^CR2^*) via CRISPR/Cas9. *Mlo8^CR1^* and *mlo8^CR2^* both showed significantly increased PM resistance on the stem and hypocotyl compared with non-transformed controls (Fig. 3). We also knocked out *CsMLO11* to obtain *mlo11^CR1^* and *mlo11^CR2^*, and generated a double mutant of *CsMLO8* and *CsMLO11* (*mlo8^CR1^mlo11^CR1^*). The *mlo11^CR1^* and *mlo11^CR2^* mutants showed significantly reduced PM infection on both stem and hypocotyl, but the resistance was comparatively weak compared with that of *mlo8^CR1^* and *mlo8^CR2^.* In contrast, the double mutant *mlo8^CR1^mlo11^CR1^* exhibited stronger resistance on the stem and hypocotyl compared with the single mutant *mol8* or *mlo11* (Fig. 3). This revealed that *CsMLO8* has a higher impact on stem PM resistance than *CsMLO11.* PM resistance in the adult and young leaf was also evaluated. Consistent with data from Tek *et al.* [41], the PM infection on *mlo8* and *mlo11* leaves was markedly decreased compared with the WT. Moreover, the PM symptoms and spore density decreased more noticeably on the stem and hypocotyls compared with the leaves, indicating that disruption of *CsMLO8* and/or *CsMLO11* results in greater reduction of PM infection on stems than on leaves.

To further illustrate the molecular mechanism of *mlo8*- and *mlo11*-mediated PM resistance, we compared the hypocotyl transcriptome of the mutants, i.e. *mlo8^CR1^*, *mlo11^CR1^*, and *mlo8^CR1^mlo11^CR1^*, with that of the WT control. The results suggested that the transcript levels of a number of genes in primary and secondary metabolic pathways were higher in *mlo8^CR1^*, *mlo11^CR1^*, and *mlo8^CR1^mlo11^CR1^* plants relative to control after pathogen inoculation; these metabolites included the aromatic amino acid l-phenylalanine, cinnamic acid, and phenylpropanoid. A diversity of secondary metabolites function in defense against biotic stress in plants [42, 43]. Aromatic amino acids (tryptophan, phenylalanine, and tyrosine) are the branch points where primary metabolites may enter secondary metabolic pathways that function in protecting plants from biotic stresses [44, 45]. Phenylpropanoids are associated with plant biotic stress tolerance [46]. In the phenylpropanoid biosynthesis pathway, l-phenylalanine is converted to *trans*-cinnamic acid to produce the precursor for the biosynthesis of salicylic acid, a crucial hormone regulating pathogen response and disease resistance [47, 48]. Moreover, several different pathways were enhanced in *mlo8^CR1^* and *mlo11^CR1^*. In *mlo8^CR1^*, cell wall organization or biogenesis pathways were enriched. Sun *et al*. [49] recently screened a PM-susceptible mutant from an EMS-mutagenized population of a natural *mlo8* mutant, and also found that PM resistance of *mlo8* was associated with increased cell wall deposition, while in *mlo11^CR1^* and *mlo8^CR1^mlo11^CR1^* defense response pathways were significantly upregulated, suggesting that *CsMLO8* and *CsMLO11* might have different defense mechanisms. Our findings are supported by the study of Tek *et al.* [41], in which *CsMLO8* was proposed as a negative regulator in the pre-invasive response, while *CsMLO11* could be associated with the post-invasive defense response.

ROS can induce damage to cellular molecules, and contribute to programmed cell death and defense response [50, 51]. The enhanced PM resistance in transgenic *Vitis vinifera* plants expressing *VqWRKY31* and *VqWRKY56* was correlated with increased levels of ROS [52, 53]. In cucumber, it was reported that PM resistance involved ethylene-mediated ROS metabolism [3, 54]. In our study, compared with the WT, a burst of ROS production was also detected in the stem of *mlo8^CR1^*, *mlo11^CR1^*, and *mlo8^CR1^mlo11^CR1^*. The ROS accumulation induced by pathogen infection was linked to calcium signaling and callose deposition at the plasmodesmata [55]. Apoplastic ROS are produced by cell wall peroxidases and Rbohs in plants [56, 57]. During pathogen invasion, the activation of pattern recognition receptors leads to apoplastic ROS production through Rbohs and peroxidase activity [57–59], and *RbohD* is the highest expressed *Rbohs* in *Arabidopsis* [58, 60, 61]. CYSTEINE-RICH RLK2 (CRK2) kinase phosphorylates the C-terminal region of RBOHD and is required for the full elicitor-induced ROS burst [40]. Here, we found that both *CsRbohD* and *CsCRK2* were more highly expressed in *mlo8^CR1^*, *mlo11^CR1^*, and *mlo8^CR1^mlo11^CR1^* (Fig. 5a). These results suggest that the loss of function of *CsMLO8* and *CsMLO11* promoted the ROS burst, contributing to PM resistance.

**Figure 8:**
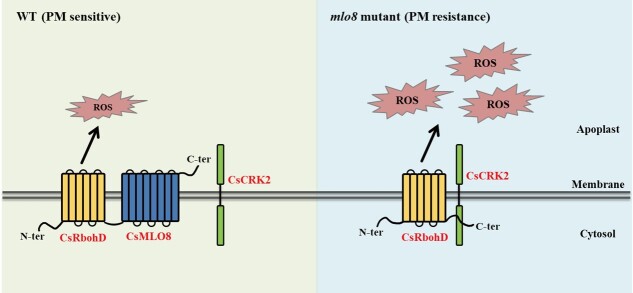
A schematic model explaining the possible mechanism of CsMLO8-triggered PM susceptibility in cucumber. CsCRK2 contributes to the activation of CsRbohD via phosphorylation of its C-terminus. In WT cucumber, CsMLO8 binds to the C-terminus of CsRbohD, resulting in inhibition of the interaction between CsCRK2 and CsRbohD; thus less ROS is produced, resulting in PM susceptibility. In an *mlo8* mutant, CsCRK2 phosphorylates the C-terminal region of CsRbohD and induces an ROS burst, resulting in the PM-resistant phenotype.

MLOs are involved in several biological processes, including root thigmomorphogenesis [62], fertilization [63], and stress response [64, 65]. The function of MLO proteins in multiple plant processes has been summarized [66], and it was proposed that MLO proteins function in responses to mechanophysical stimuli. With respect to pathogen response, barley HvMLO1 promotes arbuscular mycorrhizal colonization by *Rhizophagus. irregularis* [64] and *Serendipita. indica* infection of barley roots [65], and the development of the pathogen is terminated upon cell wall penetration in barley *mlo* mutants [67]. In *Arabidopsis*, three MLO proteins are associated with PM susceptibility: the single mutant *Atmlo2* is partially resistant and the triple mutant *mlo2mlo6mlo12* showed complete PM resistance [23]. Protein interactome analyses found that MLOs could interact with cyclic nucleotide-gated channels (CNGCs) to regulate polarized tip growth of the pollen tube [68, 69], with the vesicle-associated membrane protein VAMP72 clade to regulate calcium-dependent root thigmomorphogenesis [69], and with a cytoplasmic calcium sensor (CaM) to modulate HvMLO1-mediated PM susceptibility [70] and AtMLO10-mediated pollination [71]. Although it has been reported that MLOs regulate ROS [72, 73], the underlying molecular mechanism of how MLOs regulate the ROS signaling pathway remains unclear. In this study, our experiments demonstrated that CsMLO8 and CsMLO11 could physically interact with CsRbohD and CsCRK2. Moreover, CsMLO8 and CsCRK2 competitively interact with the C-terminus of CsRbohD. Therefore, we propose a working model to explain the possible mechanism of CsMLO8-mediated susceptibility to PM in cucumber stem (Fig. 8). In WT cucumber, CsMLO8 inhibits the interaction between CsCRK2 and the C-terminus of CsRbohD, thus less ROS is produced, resulting in PM susceptibility. In the *mlo8* mutant, the inhibition of CsCRK2 by CsMLO8 is relieved, and thus CsCRK2 is able to phosphorylate the C-terminal region of CsRbohD and induce a ROS burst, resulting in a PM-resistant phenotype. Taken together, our results revealed that CsMLO8/11 interact with CsCRK2 and CsRbohD to regulate PM resistance in cucumber stem. CsMLO8 and CsMLO11 are therefore pivotal target genes that could be manipulated to breed new cultivars with PM resistance.

## Materials and methods

### Investigation of stem powdery mildew resistance in 95 cucumber accessions

PM resistance of the adult stem was evaluated in 95 cucumber accessions collected from different geographical origins (Supplementary Data Table S1). The PM resistance of each accession was evaluated in spring 2014 and fall 2014. The experiments were conducted in the greenhouse at Changping (40°13′ N, 116°05′ E) and Shunyi farms (40°15′ N, 116°83′ E) in Beijing, China, in a randomized complete block design. Three replicates were set for each experiment, and five plants per accession were used for each replicate.

PM infection naturally occurred on the stems of adult plants 7 weeks after sowing in the greenhouse. Ten weeks after sowing, PM symptoms on the stem were ranked by disease severity of each plant on a scale of 0, 1, 3, 5, 7, or 9, based on the proportion of the stem surface with the characteristic PM spots, as follows: 0, no symptoms; 1, ≤25%; 3, 25–50%; 5, 50–75%; 7, ≥ 75%; 9, PM spots covered the entire stem. For each line, a disease index (DI) was used as an indicator to represent PM resistance in each plant. DI = 100 × ∑(number of plants with disease rating × disease rating)/(total number of plants × highest disease rating) [9].

To identify similarities and differences among the core accessions in their response to PM infection, a phylogenetic tree was constructed using SAS 9.0 based on the average DI of each accession in two seasons.

### Genome-wide association analysis

GWAS was performed with 1 702 257 SNPs using the standard mixed linear model of the software TASSEL 5.0 [74]. Manhattan plots and LD heat maps were drawn using the qqman package in the R environment [75]. The strong QTL was analyzed [−log_10_ (*P*-value) = 6.23]. SNPs in candidate genes within 200 kb of the linkage peak were identified.

### Plasmid construction and plant transformation

To generate CRISPR/Cas9 mutations in the *CsMLO8* and *CsMLO11* genes, single guide RNA (sgRNA) target sites were designed to insert into *pKSE402* with BsaI and T_4_ ligase [76]. The construct was transformed into *Agrobacterium tumefaciens* strain *EHA105*. PM-susceptible cucumber cultivar ‘CU2’, was used for transformation, referring to the previously published method [76]. Mutated plants were verified by sequencing the target sequence. T_2_ homozygous mutants of *CsMLO8* and *CsMLO11* were crossed and then selfed to screen for homozygous double mutants of *CsMLO8CsMLO11*. The oligos used are listed in Supplementary Data Table S8*.*

### Powdery mildew inoculation, disease index, and spore density

PM resistance of the *CsMLO* mutants (*mlo8^CR1^, mlo8^CR2^*, *mlo11^CR1^*, *mlo11^CR2^*, *mlo8^CR1^mlo11^CR1^*) and WT plants was evaluated at the seedling (hypocotyl, young leaf) and adult (stem, adult leaf) stages.

Experiments evaluating PM resistance at the adult stage were conducted in the greenhouse at a Changping farm (40°13′ N, 116°05′ E) in Beijing, China, in a randomized complete block design. Three replicates were set for each line, and five plants were used for each replicate. The seeds were sown on 27 July 2022. PM infection naturally occurred on the leaves ~6 weeks after sowing. PM symptoms on leaves were scored on 21 September (55 dps). PM on stems appeared ~7 weeks after sowing and was evaluated on 28 September (62 dps).

For phenotyping PM resistance at seedling stage, all lines were cultivated in an environmentally controlled growth chamber under normal conditions, with the temperature of 28/18°C (14 h day/10 h night) and 60–80% relative humidity, in a randomized complete block design. Three replicates were set for each line, and five plants were used for each replicate. The PM pathogen was collected from infected cucumber leaves in the greenhouse. When the first true leaf was fully expanded, a pathogen suspension with a concentration of 5 × 10^5^ spores/ml was sprayed evenly on the leaf and hypocotyl. PM symptoms on the first true leaf and hypocotyl were scored separately at 6 and 8 dpi.

PM symptoms on the stem, adult leaf, hypocotyl, and young leaf were ranked by disease severity based on the proportion of the surface area covered with PM spores, which was classified using the method described above for the stem. Likewise, the calculation of the DI was done in the same way [9]. At the same time, infected stem (1.0 g), hypocotyl (0.8 g), adult leaf (0.4 g), and young leaf (0.1 g) were soaked in 2 ml of sterile water with gentle agitation. Spores were counted with an automated fluorescence microscope (BX63, Olympus, Japan).

### RNA-seq analysis of transgenic cucumber plants

For RNA-seq analysis, the hypocotyl of the WT and mutants (*mlo8^CR1^*, *mlo11^CR1^*, *mlo8^CR1^mlo11^CR1^*) were sampled at 0 and 24 h after pathogen inoculation. Three independent biological replicates were used for RNA-seq experiments. Raw reads were filtered using fastp software [77]. The clean reads were then aligned to the reference genome of ChineseLong v3 (http://cucurbitgenomics.org/v2/ftp/genome/cucumber/Chinese_long/v3/) using the STAR program [78] and unique alignments were retained. DEGs between different genotypes and time-points were identified using the R DESeq2 package [79]. Genes with an absolute value of log_2_ fold change ≥2 were considered reliable DEGs. GO enrichment analysis was performed using the R clusterProfiler package [80].

### Quantitative reverse transcription PCR

Total RNA was extracted from hypocotyls using the RNeasy Plant Mini Kit (Qiagen, Hilden, Germany). qRT–PCR was performed using SYBR Premix Ex Taq™ II (TaKaRa, Kyoto, Japan). *Actin1* (*CsaV3_2G018090*) was used as the reference gene for normalization [81]. Gene relative expression level was analyzed using the 2^−ΔΔCt^ method [82]. Three biological replicates were applied. Primers used are listed in Supplementary Data Table S8.

### Reactive oxygen species and trypan blue staining of O_2_^−^ and H_2_O_2_

When the first true leaf was fully expanded, *mlo8^CR1^*, *mlo11^CR1^*, *mlo8^CR1^mlo11^CR1^*, and WT seedlings were inoculated with PM pathogen, and the stem was sampled for histochemical staining at 6 days after PM inoculation. Histochemical staining of H_2_O_2_ and O^2−^ was performed following our previously published method [83]. Young leaf was used for trypan blue staining as outlined in [52]. Six days after pathogen inoculation the first true leaf was submerged in lactophenol–trypan blue solution (30 ml ethanol, 10 ml phenol, 10 ml glycerin, 10 ml lactic acid, and 20 mg trypan blue) in glass containers, and then boiled for 1 min. Leaves were then incubated in 2.5 g ml^−1^ chloral hydrate solution for 24 h. PM incidence was observed with a microscope (BX63, Olympus, Japan) under 10× magnification.

### Yeast two-hybrid assays

Y2H assays were performed using a DUALmembrane starter kit STE (Dualsystem Biotech AG, catalog no. P01401). The coding sequence of *CsMLO8* and *CsMLO11* were separately fused to pBT3-STE as bait, and the full lengths of *CsRbohD* and *CsCRK2* were individually fused to pPR3-N as prey. Each bait and prey construct and empty vectors were paired, and transformed into yeast strain NMY51. The yeast cells were first selected on synthetic defined medium lacking leucine and tryptophan (SD−L/W), and then transferred to SD medium lacking Leu, Trp, His, and adenine (SD−L/W/H/A) to test protein interactions.

### Firefly luciferase complementation imaging assays

The LCI assay was performed following a previously described method [84]. Firstly, the coding sequences (CDSs) of *CsMLO8*, *CsMLO11*, and full-length or truncated *CsRbohD* were separately fused to *pCAMBIA1300-nLUC*, and full-length or truncated *CsRbohD* and *CsCRK2* were separately fused to *pCAMBIA1300-cLUC* vector, and the CDS of *CsMLO8* was also fused to *pCAMBIA1300-Flag*. Primers are listed in Supplementary Data Table S8. *Agrobacterium* strain GV3101 cells transformed with the nLUC and cLUC expression vectors were equally mixed and were injected into young *N. benthamiana* leaves. The LUC image was captured at 48 h after infiltration using imaging apparatus (NightSHADE LB 985, Berthold). Five independent *N. benthamiana* leaves were analyzed.

### Bimolecular fluorescence complementation assays

For BiFC analysis, the full-length CDSs of *CsMLO8*, *CsMLO11*, and *CsCRK2* were separately cloned into the *pEarleyGate201-nYFP* vector, and full-length CDSs of *CsCRK* and *CsRbohD* were separately cloned into the *pEarleyGate202-cYFP* vector. Primers are summarized in Supplementary Data Table S8. *Agrobacterium tumefaciens* strain GV3101 cells transformed with the above nYFP and cYFP constructs were equally mixed and injected into *N. benthamiana* leaves. The YFP signals were observed with a confocal microscope (Carl Zeiss, LSM880). Five independent *N. benthamiana* leaves were analyzed.

## Acknowledgements

This research was supported by the National Natural Science Foundation of China (32202487), the Beijing Municipal Science and Technology Commission Program (Z231100003723005), the Key-Area Research and Development Program of Guangdong Province (2020B020220001), the Key-Area Research and Development Program of Shandong Province (2021LZGC016), the Beijing Joint Research Program for Germplasm Innovation and New Variety Breeding (G20220628003-03), the Earmarked Fund for Modern Agro-industry Technology Research System (CARS-23), and the Science and Technology Innovation Program of the Chinese Academy of Agricultural Science (CAAS-ASTIP-IVFCAAS).

## Author contributions

S.D. drafted the manuscript; S.D., X.L., J.H., and H.M. conducted the experiments and analyzed the data; D.M.B., Y.B., J.G., X.P.L., R.Y., and X.G. helped collect the data; J.S., X.Y., and S.Z. conceived and designed the study.

## Data availability

All data that support the findings of this study are available in the article and in the supplementary materials published online.

## Conflict of interest

The authors declare that the research was conducted in the absence of any commercial or financial relationships that could be construed as a potential conflict of interest.

## Supplementary data

Supplementary data is available at *Horticulture Research* online.

## Supplementary Material

Web_Material_uhad295
