# Supplementary material for: CsMLO8/11 are required for full susceptibility of cucumber stem to powdery mildew and interact with CsCRK2 and CsRbohD
Source: Hortic Res. 2023 Dec 29;11(2):uhad295. doi: 10.1093/hr/uhad295 (PMC10894460; doi:10.1093/hr/uhad295)
Supplement: Web_Material_uhad295 [file web_material_uhad295.zip › Supplementary_file.docx]

**CsMLO8/11 are required for full susceptibility of cucumber stem to powdery mildew and interact with CsCRK2 and CsRbohD**

**Shaoyun Dong^1#^, Xin Liu^1#^, Jianan Han^1#^, Han Miao^1#^, Diane M Beckles^3^, Yuling Bai^4^, Xiaoping Liu^1^, Jiantao Guan^1^, Ruizhen Yang^2^, Xingfang Gu^1^, Jiaqiang Sun^2^*, Xueyong Yang^1^*, Shengping Zhang^1^***

*^1^State Key Laboratory of Vegetable Biobreeding, Institute of Vegetables and Flowers, Chinese Academy of Agricultural Sciences, 100081, Beijing China;*

*^2^ Institute of Crop Sciences, Chinese Academy of Agricultural Sciences, 100081, Beijing China；*

*^3^Department of Plant Sciences, University of California, Davis, One Shield Avenue, Davis, CA 95616, USA.*

*^4^Plant Breeding, Wageningen University & Research, Droevendaalsesteeg 1, 6708 PB Wageningen, The Netherlands*

*****Correspondence authors: [zhangshengping@caas.cn](mailto:zhangshengping@caas.cn); yangxueyong@caas.cn; [sunjiaqiang@caas.cn](mailto:guxingfang@caas.cn;sunjiaqiang@caas.cn).

^#^ These authors contributed equally to this work.

**
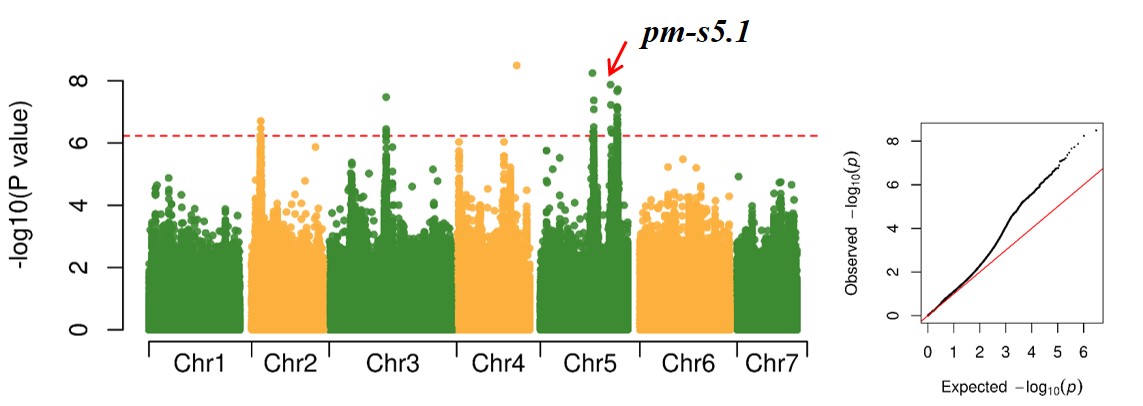
**

**Figure S1 GWAS Manhattan plots for LT tolerance in fall of 2014.** The genome-wide significance threshold was *P*=5.9×10^-7^. The red arrow indicates the position of a strong peak that was detected in two experiments (*pm-s5.1*).
